# Identifying underrepresented groups in oncology clinical trials using routinely collected data in an English academic trial setting

**DOI:** 10.1186/s13063-026-09812-2

**Published:** 2026-05-23

**Authors:** Georgiana Synesi, Rebecca Lewis, Lucy Kilburn, Judith Bliss, Emma Hall

**Affiliations:** Clinical Trials and Statistics Unit, The Institute of Cancer Research, London, SM2 5GP UK

**Keywords:** Oncology clinical trials, Demographic data, Equality, Diversity, Inclusivity

## Abstract

**Background:**

To facilitate equitable access to novel treatments, cancer trial participants should represent as far as possible those that will receive the treatment in practice. We can identify groups who rarely participate in cancer trials by collecting demographic data from participants. In the UK, there is no standardised practice around demographic data capture, leading to inconsistent collection across trials. A lack of systematically collected and published quantitative data from participants in UK cancer trials may limit our ability to identify underrepresented groups.

**Methods:**

We reviewed availability and completeness of demographic data recorded from 2235 participants in six bladder and six head and neck cancer trials conducted by the Clinical Trials and Statistics Unit at the Institute of Cancer Research (ICR-CTSU) between 2001 and 2023. To assess the representativeness of trial populations, demographic data from trial participants were compared with published data (NHS Digital) from 260,350 people who were treated for these cancers between 2013 and 2022 in England, using chi-squared goodness-of-fit tests and one-sample tests of proportion. A survey was distributed to 12 clinical trials units conducting similar trials to establish which demographic data are routinely collected across the UK.

**Results:**

Data on ethnicity, postcode, smoking status, and co-morbidity burden were inconsistently captured across ICR-CTSU trials, with missing data. Amongst the overall trial population, people older than 80 (*n* = 486/2235, 22%), females (*n* = 466/2235, 21%), people living in the most deprived areas (*n* = 390/1447, 27%), and ethnic minority groups (*n* = 5/275, 2%) were underrepresented, with some differences by treatment modality. Responses from UK trial teams showed that aside from age and sex (routinely captured), smoking status was the most consistently captured (13/17 trials).

**Conclusions:**

This study provides quantitative data on cancer trial participants examining several demographic factors and indicates potentially underrepresented groups in trials of the disease subtypes investigated. Missing data were likely observed as a result of data cleaning being focused on items directly addressing the research question. Collecting and analysing a broad range of demographic data with a focus on inclusivity can inform researchers of groups who may benefit from tailored interventions to increase accessibility to cancer trials in the future.

**Supplementary Information:**

The online version contains supplementary material available at 10.1186/s13063-026-09812-2.

## Background

A lack of diverse representation within clinical trial participant populations can limit our understanding of disease mechanisms and the generalisability of findings [1]. Recruiting a diverse group of participants can ensure that trial results are applicable to all patients who may receive the intervention once it becomes widely available. Striving to increase diversity amongst trial participants can promote equitable opportunities for participants and their communities, build trust in medical research, and generate new biomedical knowledge [2]. Demographics such as age, sex, and ethnicity may moderate the efficacy and safety profiles of drug treatments, affecting the pharmacokinetics and pharmacodynamics of pharmacological treatments [3]. Socioeconomic factors such as a person’s income or educational background have also been shown to correlate with disease prevalence and severity [4]. Despite this, several groups are thought to be underrepresented in clinical research. The National Institute for Health and Care Research Innovations in Clinical Trial Design and Delivery for the Under-served (NIHR-INCLUDE) guidance [5] identified potentially underserved groups in clinical research by demographic factors (e.g. people aged under 18 or over 75), socioeconomic factors (e.g. people living remotely), health status (e.g. people with multiple health conditions), and disease-specific factors (e.g. people with rare diseases). The guidance highlighted that whilst the definition of an underserved group may vary depending on the context, three common characteristics were:

1. “Lower inclusion in research than one would expect from population estimates”
2. “High healthcare burden that is not matched by the volume of research designed for the group”
3. “Important differences in how a group responds to or engages with healthcare interventions compared to other groups, with research neglecting to address these factors”

Researchers can monitor the inclusivity of trials by collecting demographic data from trial participants. Historically, in the UK, there has been no standard practice around collecting these data to monitor or assess inclusivity. Previously, an audit of clinical trials managed by the Clinical Trials and Statistics Unit at the Institute of Cancer Research (ICR-CTSU) found that data relating to participants’ age, ethnic group, and health status were well-collected, but data relating to socioeconomic factors were not [6]. In 2024, the DISTINCT project [7] collated a question set in collaboration with patient and public advisors to monitor inclusivity amongst trial participants based on national surveys covering protected characteristics under the Equality Act 2010 [8] and underserved groups suggested by the NIHR’s INCLUDE guidance [5]. The question set is now in the pilot phase having been implemented by a number of UK clinical trials units (CTUs) and researchers. The NIHR recently implemented a plan to collect demographic data for this purpose from grant applicants and committee members and will develop guidance on collecting these data from research participants in the future [9]. Recent recommendations are to include demographic data in published outputs where available, or to be transparent as to why they are not available [10–12].

There is a dearth of published quantitative data on the demographics of clinical trial participants in the UK. A 2017 self-reported survey investigating the engagement of Black and Asian Minority Ethnic (BAME) groups with medical research showed that respondents from UK ethnic minorities were less likely to have participated in medical research compared with White British counterparts, despite having a similar willingness to participate [13]. The NIHR conducted an audit of baseline characteristics reported in 148 randomised controlled trials (RCTs) commencing between 2007 and 2017 registered in the NIHR Journals Library [9]. The mean participant age was 51 years, and there was a relatively even spread of male and female participants. Whilst the vast majority (86%) of RCT participants were White, this was broadly in line with ethnicity data from the 2011 census. Ethnicity data was more likely to be missing and had a higher percentage of “prefer not to say” responses than other factors.

In oncology trials, complex protocols and stringent eligibility criteria can pose recruitment and retention challenges [14, 15]. In the UK between 2017 and 2021, only ~15% of those diagnosed with cancer participated in a clinical trial on average each year [16–18]. A 2010 case study of oncology trials at an English hospital trust showed that the odds of being in a trial were 30% lower for a patient from a minority ethnic background compared with a White patient [19]. The authors reported a lack of available data on ethnicity and cancer prevalence amongst people according to their ethnicity as a limitation. To our knowledge, these are the most up-to-date demographic data from UK oncology trial participants available which have been published with the aim of assessing inclusivity of trials.

Bladder and head and neck cancers are the 11th and 8th most commonly diagnosed cancers respectively each year in the UK [20]. The incident populations of these cancers share characteristics in terms of demographic data and lifestyle factors (Table 1). These characteristics, and the ways in which they interact, may influence clinical trial participation.

**Table 1:** Summary of incident population characteristics

Factor	Relationship with incidence	Bladder cancer cases	Head and neck cancer cases	References
Age	Higher incidence in older age groups	56% diagnosed in people aged 75 and older	36% diagnosed in people aged 75 and older	[21, 22]
Sex	Higher incidence in males	73% diagnosed in males	69% diagnosed in males	
Deprivation	Higher incidence in socioeconomically disadvantaged groups	Diagnoses 47% higher in disadvantaged vs non-disadvantaged males	Diagnoses 101% higher in disadvantaged vs non-disadvantaged males	
		Diagnoses 23% higher in disadvantaged vs non-disadvantaged females	Diagnoses 64% higher in disadvantaged vs non-disadvantaged females	
Ethnicity	Predominantly diagnosed in White ethnic groups	93% diagnosed in people from White ethnic groups	90% diagnosed in people from White ethnic groups	[23]
Smoking status	Known risk factor	45% attributed to smoking tobacco	36% attributed to smoking tobacco	[24]
Co-morbidity burden	Higher rates of co-morbid conditions: Hypertension (30% UK population) Diabetes mellitus (10% UK population)	Co-incidence of hypertension reported as high as 77%, and diabetes mellitus 27%	Co-incidence of hypertension reported as high as 60%, and diabetes mellitus 21%	[25–28]

The ICR-CTSU has a large portfolio of national, multi-centre, RCTs. ICR-CTSU bladder cancer trials investigate chemotherapy, immunotherapy, radiotherapy, and surgical treatment, whilst trials for head and neck cancer focus on radiotherapy. Typically, around 1000 patients are enrolled in ICR-CTSU managed trials at over 100 cancer centres and regional hospitals across the UK and internationally each year. The trial portfolio includes around 70 active trials at different stages from set-up to reporting. Any demographic data included in trials’ case report forms (CRFs) are completed by clinicians and research staff at participating sites, rather than self-report by participants [6]. Given the lack of information available about UK trial participants, we felt it would be valuable to assess the demographic data from participants in ICR-CTSU bladder or head and neck oncology trials. The aims of this project were to assess the completeness of demographic data collection in ICR-CTSU trials, to use readily available population-level data to identify underrepresented groups amongst ICR-CTSU trial participants, and to establish which demographic data are routinely captured by CTUs conducting non-commercial bladder and head and neck oncology trials in an academic setting.

## Methods

Eligible studies were bladder or head and neck oncology RCTs which were managed by ICR-CTSU from initiation and had completed recruitment. This included studies recruiting between 2001 and 2023. Although ICR-CTSU trials enrol from sites in England, Northern Ireland, Scotland, Wales, Australia, and New Zealand, the comparator data referred only to patients treated in England. Participants recruited from non-English sites therefore were not considered in the statistical analysis. Data from participants in ICR-CTSU trials (the trial population) were compared with aggregate data from the English population who received treatment for bladder or head and neck cancer between 2013 and 2022, published by NHS Digital [29] (the treated population).

Demographic and lifestyle factors of interest were age, sex, ethnicity, postcode (as a measure of deprivation), smoking status, and co-morbidity burden. These were selected due to their relationship with incidence, or their roles as risk factors for the development of the chosen cancers (Table 1), and in relation to demographic data typically captured across ICR-CTSU trials. Where relevant data were captured, Charlson Comorbidity Index (CCI) scores were used to assess co-morbidity burden. The CCI predicts 10-year survival in patients with multiple morbidities where a higher score indicates an individual has more co-morbid conditions [30]. For trials which did not capture CCI, we chose to assess co-morbidity burden related to hypertension and diabetes mellitus, due to their association with the cancers being investigated (Table 1). Postcodes were allocated using an online tool [31] to a quintile from most to least deprived based on the English indices of deprivation which accounts for income, employment, education, health, crime, barriers to housing and services, and living environment. Whilst this tool cannot be used to measure an individual’s deprivation, it can give an area-based measure of relative deprivation [32].

Data from trials investigating the same treatment modality were combined for comparison with data from the treated population. Combining trials in this way allowed ICR-CTSU data to be compared with those from an equivalent population who were treated with each modality. We conducted a sensitivity analysis comparing demographic data from the trial and treated populations without separating data by treatment modality, and did not find major differences in the results. Data from ICR-CTSU bladder cancer trials were compared with data from the English population treated with systemic anti-cancer therapy (SACT) in combination with radiotherapy (RT) (referred to as chemoradiotherapy) or tumour resection. For trials investigating non-muscle-invasive bladder cancers (NMIBC), weighted averages of demographic data from the English treated population who had their tumours resected alone or in combination with other treatment were calculated and used for statistical analyses. For trials investigating muscle-invasive bladder cancers (MIBC), weighted averages of data from people who received SACT only, RT only, and chemoradiotherapy were calculated.

**Fig. 1:**
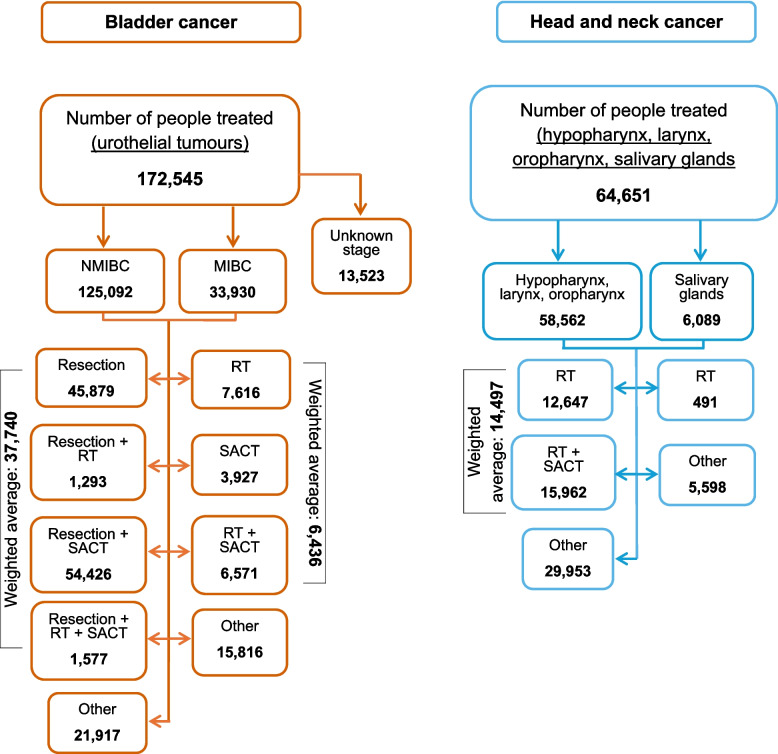
The number of patients treated for bladder or head and neck cancer between 2013 and 2022 in England with modalities used in ICR-CTSU trials. “RT + SACT” refers to combined treatment: chemoradiotherapy. “Other” treatment includes SACT only, RT only, and other care (NMIBC), any therapies including resection and other care (MIBC), and SACT only, any therapies including resection, and other care (head and neck cancers). Other care is defined as treatments other than RT, SACT or resection, treatment outside the assessed timeframe, treatment in a private care setting, or where there are missing data [29]

Data from ICR-CTSU head and neck cancer trials were compared with data from the English population treated with chemoradiotherapy, or RT alone. As patients in the English treated population receiving chemoradiotherapy would also have been eligible to receive RT, weighted averages of data from people treated with these therapies were calculated and used for statistical analyses [29]. Trials investigating chemoradiotherapy included hypopharyngeal, laryngeal, and oropharyngeal tumours, so data from the population treated for tumours of each anatomical subsite were amalgamated for comparison with the trial population. All trials investigating RT alone were for parotid tumours, so only data from the population treated for major salivary gland tumours were compared with the trial population for this modality (see Fig. 1).

Raw data from the trial population were categorised according to the published categories from the treated population data [29] (Table 3). Trial participant ages were grouped as under 50, 50–59, 60–69, 70–79, and over 80. Ethnicity was categorised as Asian, Black, Other, Unknown, or White, where “Other” includes mixed and multiple ethnic groups [33]. In instances where numbers in each broad ethnic group were too small to draw meaningful conclusions, categories were combined as “additional groups” for statistical analysis [34]. Where relevant data were captured, Charlson Comorbidity Index (CCI) scores were calculated by the authors for individual participants, then summarised for the cohort. Data on hypertension and diabetes mellitus as co-morbidities were extracted and analysed narratively, by counting the number recorded in free-text fields on the case report form.

### Statistical methods

Data cleaning and analysis was conducted using Stata version 17 and Microsoft Excel. Descriptive statistics were used to assess data where appropriate. The availability of demographic data was assessed by measuring the number of trials capturing each demographic factor, and the amount of missing data if applicable. Trials which did not capture particular demographic data factors were omitted from the analysis of those factors, rather than being considered missing data. Chi-squared goodness-of-fit tests were used to compare differences in age group, sex, ethnicity, and deprivation quintile between the trial and treated populations. A *p *value of 0.01 was considered statistically significant to make some account for multiple testing. One-sample tests of proportion were used to compare the proportion of current smokers amongst the trial population with the percentage of cancer cases that are attributable to smoking [24]. Data on co-morbidity burden were analysed narratively.

### Survey to CTUs

To establish a pattern of demographic data collection amongst UK CTUs conducting academic trials, an online survey was developed and distributed using Jisc version 3 (Appendix 2). The survey asked about capturing data on protected characteristics under the Equality Act 2010 [8], caregiving responsibilities, and measures of socioeconomic status. The survey was distributed to CTUs managing trials which matched the following criteria, to reflect those conducted at the ICR-CTSU:

- Interventional, aimed at assessing the efficacy or safety of treatments
- For bladder cancer (ICD-10 C67) or head and neck cancers (ICD-10 C00–14, C30–32)
- Phase II or III, multi-centre, randomised controlled trials with at least one site in the UK
- Recruited patients between 2013 and 2023

To identify eligible trials, a comprehensive search was conducted between February and March 2023 using ClinicalTrials.gov, ISRCTN registry, and NIHR Clinical Research Network portfolio, to capture clinical trials of IMPs (CTIMPs) and trials evaluating other (non-IMP) therapies. For CTUs at which an author had a known contact, a preliminary email was sent to explain the purpose of the survey and to find an appropriate respondent. For the remaining CTUs, the survey was distributed to specific trial team emails where available. Where trial specific emails were not available, the survey was sent to the listed contact on the trial registration page. Two reminders were sent to contacts by email. The survey was opened in February 2024 and closed after 2 months. Responses were analysed narratively, with the number of trials capturing each demographic factor counted and reported along with any missing data if applicable.

## Results

### Trial characteristics and participants

Six bladder cancer trials and six head and neck cancer trials were eligible for this project (Appendix 1). A total of 2457 participants were recruited (1466 in bladder trials and 991 in head and neck trials). The number of participants residing in England (data used for analyses) was 2235/2457 (91%). Table 2 shows how ICR-CTSU trials were split by treatment modality.

**Table 2:** The number of ICR-CTSU trials and participants residing in England split by treatment modality. “RT + SACT” refers to combined treatment: chemoradiotherapy. “NMIBC” refers to non-muscle-invasive bladder cancer, and “MIBC” refers to muscle-invasive bladder cancer

ICR-CTSU trials	Treatment modality used in trial				
	RT *n* trials (*n* participants)	Resection *n* trials (*n* participants)	RT + SACT *n* trials (*n* participants)	RT + SACT + resection *n* trials (*n* participants)	Total (participants)
NMIBC	–	Two (544)	–	–	544
MIBC	–	–	Three (793)	One (45)	838
Head and neck	Two (191)	–	Four (662)	–	853
Total (participants)	191	544	1455	45	2235

Data from the treated population, published by NHS Digital [29], revealed that there were 172,545 people treated for urothelial bladder tumours and 64,651 people treated for hypopharyngeal, laryngeal, oropharyngeal, or salivary gland tumours between 2013 and 2022. Figure 1 shows the number of people receiving each treatment modality.

### Availability of demographic data

**Fig. 2:**
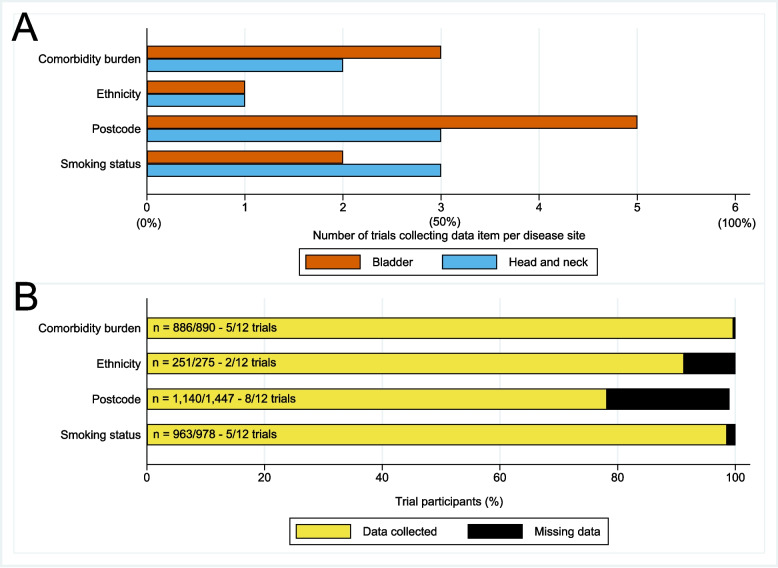
**A** Number of trials per disease subtype recording data on each demographic factor of interest (bladder trials *n* = 6, head and neck trials *n* = 6). **B** Amount of missing data amongst trials recording each demographic factor of interest. *n* = number of participants data collected from/total number of participants across all trials capturing data item

Age and sex were captured in every trial and collected from all ICR-CTSU participants. Figure 2 shows the number of trials recording other demographic data, and the amount of missing data for each item. Trials which did not capture data items have not been considered as having missing data.

### Comparison of ICR-CTSU and comparator data

People aged 70 and older were underrepresented in all trials, except for MIBC chemoradiotherapy trials, where fewer people aged 80 and older were enrolled than expected (Table 3). Whilst female participants were underrepresented in chemoradiotherapy trials across disease sites, there were no significant differences for other treatment modalities (Table 3).

**Table 3:** Chi-squared goodness-of-fit analyses, comparing data from ICR-CTSU trial population to the treated population [29]. In ICR-CTSU trial population data, *n* = number and % = proportion of participants in each category. In treated population data, % = proportion of tumours treated in each category. “Not collected” includes participants in trials which did not capture the item of interest, and missing data from participants in trials that did. Percentages may not sum to 100% across groups due to rounding error

Cancer	Treatment	Demographic factor	ICR-CTSU trial population		England treated population	*p* value
			*n*	%	%	
Bladder cancer (urothelial, known stage) Trial population *n* = 1382 Treated population *n* (raw) = 159,022	NMIBC: resection Trial population *n*: 544 Treated population *n* (raw): 103,598 Treated population *n* (weighted average): 37,740	**Age group**				
		Under 50	26	4.8	3.8	<0.001
		50–59	79	14.5	9.3	
		60–69	218	40.1	22.5	
		70–79	164	30.2	36.9	
		80+	57	10.5	27.4	
		Total collected	544	100%	100%	
		Not collected	0	–	–	
		**Biological sex**				
		Male	429	78.9	76.9	0.278
		Female	115	21.1	23.1	
		Total collected	544	100%	100%	
		Not collected	0	–	–	
		**IMD quintile**				
		1	33	12.5	16.5	0.599
		2	42	15.9	18.1	
		3	49	18.6	20.9	
		4	66	25.0	22.5	
		5	74	28.0	22.0	
		Total collected	264	100%	100%	
		Not collected	280/544	51.5%	–	
		**Broad ethnic group**				
		White	79	96.3	91.5	0.120
		Additional groups	3	3.7	8.5	
		Total collected	82	100%	100%	
		Not collected	462/544	84.9%	–	
	MIBC: RT + SACT (chemoradiotherapy) Trial population *n* = 838 Treated population *n* (raw) = 18,114 Treated population *n* (weighted average): 6436	**Age group**				
		Under 50	9	1.1	1.5	<0.001
		50–59	65	8.2	5.4	
		60–69	192	24.2	17.8	
		70–79	348	43.9	37.2	
		80+	179	22.6	38.1	
		Total collected	793	100%	100%	
		Not collected	0	–	–	
		**Biological sex**				
		Male	634	80.0	73.8	<0.001
		Female	159	20.0	26.2	
		Total collected	793	100%	100%	
		Not collected	0	–	–	
		**IMD quintile**				
		1	50	14.3	17.9	<0.001
		2	60	17.2	18.9	
		3	59	16.9	21.1	
		4	95	27.2	21.0	
		5	85	24.4	21.4	
		Total collected	349	100%	100%	
		Not collected	444/793	56.0%	–	
	MIBC: RT + SACT + resection Trial population *n* = 45 Treated population *n* (raw) = 24,816 Treated population *n* (weighted average): 5452	**Age group**				
		Under 50	1	2.2	1.8	<0.001
		50–59	6	13.3	6.4	
		60–69	26	57.8	19.7	
		70–79	11	24.4	37.9	
		80+	1	2.2	34.2	
		Total collected	45	100%	100%	
		Not collected	0	–	–	
		**Biological sex**				
		Male	40	88.9	74.1	0.020
		Female	5	11.1	25.9	
		Total collected	45	100%	100%	
		Not collected	0	–	–	
		**IMD quintile**				
		1	5	12.5	17.6	0.002
		2	12	30.0	18.7	
		3	1	2.5	21.1	
		4	6	15.0	21.5	
		5	16	40.0	21.1	
		Total collected	40	100%	100%	
		Not collected	5/45	11.1%	–	
Head and neck cancer (all anatomical subsites) Trial population *n* = 853 Treated population *n* (raw) = 29,100	RT + SACT (chemoradiotherapy) Trial population *n* = 66 Treated population *n* (raw) = 28,609 Treated population (weighted average): 14,497	**Age group**				
		Under 50	85	12.9	8.6	<0.001
		50–59	262	39.6	29.4	
		60–69	250	37.8	35.6	
		70–79	60	9.1	20.2	
		80+	5	0.8	6.2	
		Total collected	662	100%	100%	
		Not collected	0	–	–	
		**Biological sex**				
		Male	547	82.6	78.2	0.006
		Female	115	17.4	21.8	
		Total collected	662	100%	100%	
		Not collected	0	–	–	
		**IMD quintile**				
		1	107	22.0	24.7	0.094
		2	88	18.1	20.4	
		3	110	22.6	19.7	
		4	85	17.5	18.5	
		5	97	19.9	16.8	
		Total collected	487	100%	100%	
		Not collected	175/662	26.4%	–	
		**Broad ethnic group**				
		White	167	98.8	92.5	0.002
		Additional groups	2	1.2	7.5	
		Total collected	169	100%	100%	
		Not collected	493/662	74.5%	–	
	RT only Trial population *n* = 191 Treated population *n* = 491	**Age group**				
		Under 50	45	23.6	6.1	<0.001
		50–59	59	30.9	7.3	
		60–69	48	25.1	14.7	
		70–79	32	16.8	27.5	
		80+	7	3.7	44.4	
		Total collected	191	100%	100%	
		Not collected	0	–	–	
		**Biological sex**				
		Male	119	62.3	64.2	0.585
		Female	72	37.7	35.8	
		Total collected	191	100%	100%	
		Not collected	0	–	–	

Comparisons of data relating to ethnicity, deprivation, smoking status, and co-morbidity burden refer only to trials where demographic factors were captured. The vast majority of people in the trial and treated populations were from White ethnic groups (Table 3), with minority ethnic groups being underrepresented in head and neck chemoradiotherapy trials (*p* = 0.002). People living in the most deprived quintiles were underrepresented in all MIBC trials (*p* < 0.01). There were no significant differences in distribution of people living in each deprivation quintile observed amongst NMIBC and head and neck cancer trial participants (Table 3).

Of bladder cancer trials, smoking status was only captured in those for NMIBC, with missing data from 6/544 (1.1%) participants. Of participants with data available, 81/538 were smokers (15.1%), 287/538 smoked previously (53.3%), and 170/538 had never smoked (31.6%). There were significantly fewer current smokers than expected in bladder cancer trials (Table 1). Of head and neck cancer trials, smoking status was only captured in trials investigating chemoradiotherapy, with missing data from 9/434 participants. Of participants with data available, 169/425 were current smokers (39.8%), 45/425 smoked previously (10.6%), and 211/425 had never smoked (49.6%). There was no significant difference in the proportion of current smokers in head and neck trials compared with the expected proportion (Table 1).

Varied data on co-morbidity burden was captured from 602 participants in bladder cancer trials. Two trials recorded data on hypertension, with 176/544 (32.4%) participants reporting it. Three trials recorded data on diabetes mellitus, with 67/602 (11.1%) participants reporting it. One radiotherapy trial, HYBRID, captured co-morbidity burden according to the CCI. However, the scores were not calculated by the original trial statistician. We found that the average CCI score amongst HYBRID participants was seven, and all participants had CCI scores of 3+, compared with just 13% of people in the treated population [29]. Amongst reporting head and neck cancer trials, 64/284 participants (22.5%) had hypertension, and 26/284 (9.2%) had diabetes mellitus.

### Survey to CTUs

Excluding the ICR-CTSU trials involved in this audit, 19 trials were eligible for the survey, which was sent to the 12 CTUs responsible. Responses were received from 11/12 CTUs, regarding 17/19 trials (89.4%). Of these, 12/17 were bladder cancer trials and 5/17 were head and neck cancer trials.

**Fig. 3:**
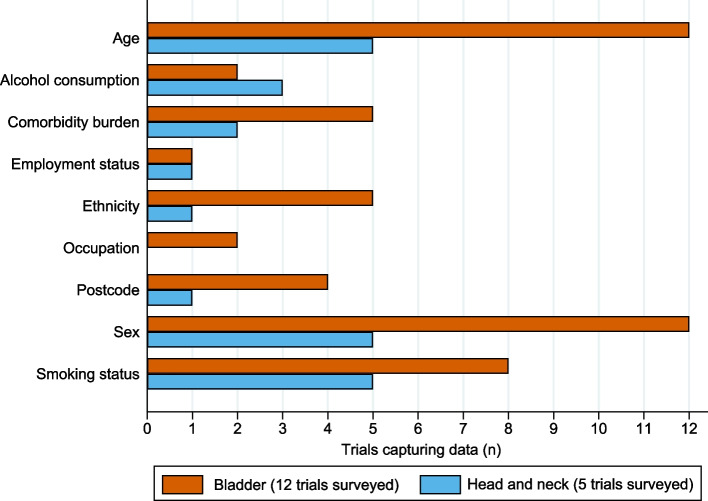
Survey results showing the number of bladder trials (total surveyed: 12) and head and neck trials (total surveyed: 5) across the UK collecting data on demographic and lifestyle factors from participants

Age and sex were captured in 17/17 of the trials surveyed. Gender identity, sexual orientation, caregiving responsibilities, religion, language preferences, income, and educational qualifications were not captured by any of the trials surveyed, in common with trials at ICR-CTSU. Figure 3 shows the number of trials which captured each item. Aside from age and sex, data on smoking status was the most consistently recorded across CTUs (13/17, 76.4%). Free-text survey responses justified the capture of smoking status as a relevant risk factor, impacting prognosis and side effect burden.

## Discussion

Not all demographic factors of interest for monitoring inclusivity were captured in every ICR-CTSU trial, and in some cases, there were missing data in trials which did record these factors. This is likely due to data cleaning being focused on items directly addressing the research question, or those which are clinically relevant. This was reflected in the survey results from other UK CTUs, where the most broadly captured data were smoking status and co-morbidity burden. Given recent guidelines and recognition of the importance of EDI by funders and research organisations [7, 9–12], one may expect increased demographic data capture in the future. Reflecting this, we found that trials designed more recently tended to capture a broader range of demographic data, with the ICR-CTSU TORPEdO head and neck trial capturing additional data on education, employment, marital status, and caregiving responsibilities. At the ICR-CTSU, the demographic data questionnaire developed through the DISTINCT project [7] will be used in all new trials, which captures these factors. DISTINCT is also being piloted in trials in a range of disease settings across the UK. Capturing a broader range of demographic data can indicate potentially underserved groups that trial teams may not have otherwise considered. This can inform the development of targeted recruitment interventions with the aim of increasing accessibility to and therefore inclusivity of trials.

Patients aged 70 and older were underrepresented in the ICR-CTSU trial population compared with the treated population across several disease sites and treatment modalities. A US study found that people aged 65 and older were often ineligible for oncology trials based on their medical histories and protocol-defined exclusionary criteria [35]. In line with this, a systematic review identified stringent eligibility criteria as a barrier to this group, along with reluctance from doctors to enrol them due to concerns around safety, and a lack of awareness of these patients about opportunities for trials [36]. Despite this, the average age of participant in the ICR-CTSU HYBRID bladder cancer trial was 83 years, and people aged 80 and older were overrepresented compared with the treated population. HYBRID was designed to include patients unfit for daily radiotherapy and serves as an example of how to design research to include an underrepresented group of patients.

Females were underrepresented in bladder chemoradiotherapy trials, broadly in line with research from the USA showing that 27% of the US bladder cancer population were female, whilst comprising 20% of clinical trial participants [37]. Although there is a higher incidence of bladder cancer amongst males, females have worse clinical outcomes [38]. Focusing efforts on increasing recruitment amongst females may help to diminish disparities in clinical outcomes. Whilst females were also underrepresented in head and neck chemoradiotherapy trials, nearly 40% of participants in head and neck radiotherapy trials were female. Given that these trials included participants with parotid tumours only, this finding may be explained by the fact that 47% of parotid tumours are diagnosed in females, compared with just 31% amongst other anatomical subsites [29]. As previously highlighted by Patel et al. [6], there was a lack of consistency in sex and gender nomenclature across trials. Given that the trials included in this audit recruited between 2001 and 2023, this finding may reflect a lack of available guidance on sex and gender nomenclature at the time of developing data capture forms. To address this challenge, ICR-CTSU templates and guidance have been updated to clarify whether data capture refers to sex or gender identity [6].

Ethnic minority groups were underrepresented in the ICR-CTSU head and neck trial population compared with the UK treated population. In England, the incidence of these cancers is significantly lower in people from ethnic minority groups compared with people from White ethnic groups [23]. However, whilst people from Asian ethnic groups comprise 2.5% of the incident population across all cancer types, they comprise 3.5% of the incident population of head and neck cancers [23]. Considering intersectionality, women from Asian ethnic backgrounds have a higher incidence of oral cancers than women from White ethnic backgrounds [39]. These disparities may reflect a lower uptake of cancer screening and subsequently later-stage diagnoses, as a result of poorer awareness of symptoms [39]. A study highlighting barriers to people from ethnic minority backgrounds accessing healthcare categorised them as extrinsic or organisational (e.g. location of health services and lack of staff training) and intrinsic or personal (e.g. language barriers and cultural differences) [40]. A multi-faceted approach from sponsors designing trials to address some of these barriers may improve the recruitment of ethnic minority groups to oncology trials. Ethnicity data were recorded in just 2/12 ICR-CTSU and 6/17 other CTU trials. These findings are in line with a literature review of high-impact medical journals published between 2020 and 2022, where ethnicity was reported in just 35/100 eligible articles. This may reflect a historic lack of standardisation in capturing and reporting demographic data for the purpose of monitoring inclusivity [41]. At the ICR-CTSU, ethnicity data are now routinely collected from trial participants [7]. Inaccuracies and inconsistencies in collecting and reporting these data may also be improved by allowing participants to self-report ethnicity [42].

People living in the most deprived areas of the UK were underrepresented in ICR-CTSU MIBC trials compared with the treated population. Previous research has shown a negative correlation between income and clinical trial participation [43]. Living rurally may also reduce accessibility to clinical trials. A polling study of low-income New York City residents showed that 25% of respondents had missed a clinical appointment due to a lack of transport options [44]. Given that clinical trial participation often requires more clinic visits than standard of care treatment, people living in more deprived areas may have reduced access. A higher level of deprivation may also affect clinical outcomes, and it was shown that compared with trial participants living in the most affluent areas, those in the least affluent areas had lower rates of overall, progression-free, and cancer-specific survival [45]. Regarding early-phase cancer trials in the UK, patients living in the least deprived areas were twice as likely to be referred compared with patients living in the most deprived areas [46, 47]. These data suggest that sponsors should consider methods of supporting socioeconomically disadvantaged populations to access clinical research. However, the NIHR and Health Research Authority do not mandate reimbursement of participation expenses [48, 49]. One ICR-CTSU head and neck trial included in this study, TORPEdO, offered reimbursement of travel and accommodation costs for participants receiving proton beam therapy (PBT), as only two centres in the UK offer PBT. ICR-CTSU standard operating procedures have been updated to ensure reimbursement for additional visits is considered during the trial budgeting process.

The underrepresentation of current tobacco-smokers in ICR-CTSU bladder cancer trials could reflect the likelihood of current smokers having a higher co-morbidity burden and therefore being more likely to be excluded by protocol-defined criteria. A limitation with this analysis was that the comparator data did not account for people who previously used tobacco, but this group comprised the majority of ICR-CTSU trial participants these data were collected from.

The incidence of hypertension and diabetes mellitus amongst ICR-CTSU trial participants was markedly lower than estimates from the literature amongst those with bladder or head and neck cancers in the general population [25–27]. The lower co-morbidity burden in the trial population compared with the incident population may reflect safety concerns with including patients with multiple morbidities in clinical trials. However, the higher CCI scores amongst participants in the HYBRID bladder cancer trial highlights the feasibility of including this population in clinical trials when studies are designed to consider them. Whilst outside the scope of the current paper, the authors have investigated the underrepresentation of typically underserved groups in relation to co-morbidity burden across UK trials in a recently published scoping review [50].

Data from the treated population were aggregated, meaning that we did not have access to individual patient data and were not able to cross-tabulate demographic factors to conduct multivariate analyses. In the future, it would be useful to use individual patient data to consider the impact of intersectionality of several demographic and lifestyle factors on trial participation.

### Limitations

Data from trial participants was compared with data from the treated population. Whilst this was not a like-for-like comparison, it gives an indication of the inclusivity of ICR-CTSU trials in comparison to national data. There were some limitations to the comparator data. It is unclear whether patients who were treated with several treatment modalities, e.g. SACT and RT, were given chemoradiotherapy, or whether they separately received SACT and RT within the assessed timeframe (18 months). It is also unclear whether patients who received several modalities separately were included in multiple datasets, or whether they were classed as having one treatment only. Finally, amalgamating data from individual anatomical subsites of head and neck cancers for ease of comparison with the trial population may have masked any demographic differences between the populations diagnosed with tumours in each subsite. These discrepancies may lead to uncertainties in the ways the treated population data were assessed, and therefore the ways in which the results were interpreted.

Due to the broad time periods of trials investigated in this study, in some older ICR-CTSU head and neck RT trials, concurrent SACT was not permitted. These trials were classed as RT trials and compared to the equivalent treated population. However, given that standard of care treatment is now chemoradiotherapy, the influence of demographic data (particularly age) on trial participation may have been underestimated. Finally, whilst ICR-CTSU data were from trials recruiting between 2001 and 2023, the comparator data from the treated population were from 2013 to 2022. However, the ICR-CTSU trial recruitment period was skewed by including a handful of older trials; 8/12 trials recruited participants between 2013 and 2022.

## Conclusion

To our knowledge, whilst many trial teams now publish the age and sex of participants, this study provides the first quantitative data on bladder and head and neck cancer trial participants which examines additional demographic factors in comparison to the wider population. These data identify potentially underserved groups in non-commercial bladder and head and neck cancer trials conducted in an academic setting. Importantly, the findings indicate potentially underrepresented groups to explore in other disease settings. The results of the survey also indicate current practice of demographic data capture in the investigated trials across the UK, and the ways in which inclusivity monitoring could be improved. Collecting and analysing a broad range of demographic data can enable underserved groups to be identified, whilst also monitoring their retention and ensuring support throughout the trial process. Aligning with recent NIHR inclusion guidance [5], progress in this area can facilitate the development of successful recruitment interventions to increase accessibility to oncology trials in the future.

## Supplementary Information

Supplementary Material 1.

Supplementary Material 2.

Supplementary Material 3.

## Data Availability

The datasets used and/or analysed during the current study are available from the corresponding author on reasonable request.

## Abbreviations

BAME: Black and Asian Minority Ethnic
CCI: Charlson Comorbidity Index
CRF: Case report form
CTU: Clinical trial unit
ICR-CTSU: Clinical Trials and Statistics Unit at the Institute of Cancer Research
IMD: Indices of Multiple Deprivation
INCLUDE: Innovations in Clinical Trial Design and Delivery for the Under-served
ISRCTN: International Standard Randomised Controlled Trial Number
MIBC: Muscle-invasive bladder cancer
NHS: National Health Service
NIHR: National Institute for Health and Care Research
NMIBC: Non-muscle-invasive bladder cancer
RCT: Randomised controlled trial
RT: Radiotherapy
SACT: Systemic anti-cancer therapy

## References

[1] Arias F, Rogus-Pulia N, Kind AJH. Improving representativeness in clinical trials and research: facilitators to recruitment and retention of underrepresented groups. Washington (DC): National Academies Press (US); 2022.

[2] Schwartz AL, Alsan M, Morris AA, Halpern SD. Why diverse clinical trial participation matters. N Engl J Med. 2023;388(14):1252–4. 37017480 10.1056/NEJMp2215609

[3] Gross AS, Harry AC, Clifton CS, Della Pasqua O. Clinical trial diversity: an opportunity for improved insight into the determinants of variability in drug response. Br J Clin Pharmacol. 2022;88(6):2700–17. 35088432 10.1111/bcp.15242 PMC9306578

[4] Mackenbach JP, Kulhánová I, Bopp M, Deboosere P, Eikemo TA, Hoffmann R, et al. Variations in the relation between education and cause-specific mortality in 19 European populations: a test of the “fundamental causes” theory of social inequalities in health. Soc Sci Med. 2015;127:51–62. 24932917 10.1016/j.socscimed.2014.05.021

[5] NIHR. Improving inclusion of under-served groups in clinical research: guidance from INCLUDE project 2020 [Available from: https://www.nihr.ac.uk/documents/improving-inclusion-of-under-served-groups-in-clinical-research-guidance-from-include-project/25435.

[6] Patel D, Kilburn L, Fox L, Hall E, Bliss J, Lewis R. Equality, diversity, and inclusion in oncology clinical trials: an audit of essential documents and data collection against INCLUDE under-served groups in a UK academic trial setting. BMC Med Ethics. 2023;24(1):105. 38017503 10.1186/s12910-023-00987-w PMC10685485

[7] Lidington E, Stiles M, Maudsley J, Ching J, Deutsch A, Lipman N, et al. Establishing a set of acceptable demographic questions for use in health research through public consultation. Res Involv Engagem. 2026;12(1). 10.1186/s40900-026-00836-1. 41606646 10.1186/s40900-026-00836-1 PMC12924441

[8] Gov.UK. Equality Act 2010. 2010. from https://www.legislation.gov.uk/ukpga/2010/15/contents.

[9] NIHR. Randomised controlled trial participants: diversity data report: NIHR; 2022 [Available from: https://www.nihr.ac.uk/documents/randomised-controlled-trial-participants-diversity-data-report/31969#report-highlights.

[10] MESSAGE Writing Group, Witt A, Woodward M, Jenkins K, Jenkins B, Hockham C. Accounting for sex and gender in biomedical, health and care research: a policy framework for UK research funders. Medical Science Sex and Gender Equity; 2024.

[11] Flanagin A, Frey T, Christiansen SL, Committee AMoS. Updated guidance on the reporting of race and ethnicity in medical and science journals. JAMA. 2021;326(7):621–7. 34402850 10.1001/jama.2021.13304

[12] Flanagin A, Frey T, Christiansen S, Bibbins-Domingo K. Draft guidance on reporting gender, sex, gender identity, sexual orientation, and age in medical and scientific publication—call for review and comment. JAMA. 2024;332(8):e2416646-e. 39190118 10.1001/jama.2024.16646

[13] Smart A, Harrison E. The under-representation of minority ethnic groups in UK medical research. Ethn Health. 2017;22(1):65–82. 27174778 10.1080/13557858.2016.1182126

[14] Kumar G, Chaudhary P, Quinn A, Su D. Barriers for cancer clinical trial enrollment: a qualitative study of the perspectives of healthcare providers. Contemp Clin Trials Commun. 2022;28:100939. 35707483 10.1016/j.conctc.2022.100939 PMC9189774

[15] Kim ES, Bruinooge SS, Roberts S, Ison G, Lin NU, Gore L, et al. Broadening eligibility criteria to make clinical trials more representative: American Society of Clinical Oncology and Friends of Cancer Research joint research statement. J Clin Oncol. 2017;35(33):3737–44. 28968170 10.1200/JCO.2017.73.7916 PMC5692724

[16] Clinical trials in cancer: barriers in access to clinical trials, especially in light of the COVID-19 pandemic. The Institute of Cancer Research; 2021. from https://www.icr.ac.uk/docs/default-source/migrated-documents/corporate-docs--accounts--and-annual-reports/icr-report-clinical-trials-in-cancer.pdf?sfvrsn=d6a98401_2.

[17] Estimates of the population for the UK, England, Wales, Scotland and Northern Ireland: The Office of National Statistics; 2017–2021 [Available from: https://www.ons.gov.uk/peoplepopulationandcommunity/populationandmigration/populationestimates/datasets/populationestimatesforukenglandandwalesscotlandandnorthernireland.

[18] Cancer registration statistics, England: NHS Digital; 2022 [Available from: https://digital.nhs.uk/data-and-information/publications/statistical/cancer-registration-statistics.

[19] Godden S, Ambler G, Pollock AM. Recruitment of minority ethnic groups into clinical cancer research trials to assess adherence to the principles of the Department of Health Research Governance Framework: national sources of data and general issues arising from a study in one hospital trust in England. J Med Ethics. 2010;36(6):358–62. 20439335 10.1136/jme.2009.033845

[20] Cancer incidence for common cancers: Cancer Research UK; 2018 [Available from: https://www.cancerresearchuk.org/health-professional/cancer-statistics/incidence/common-cancers-compared#heading-Zero.

[21] Bladder cancer incidence statistics: Cancer Research UK; [Available from: https://www.cancerresearchuk.org/health-professional/cancer-statistics/statistics-by-cancer-type/bladder-cancer/incidence#heading-Zero.

[22] Head and neck cancers incidence statistics: Cancer Research UK; [Available from: https://www.cancerresearchuk.org/health-professional/cancer-statistics/statistics-by-cancer-type/head-and-neck-cancers/incidence#heading-Zero.

[23] Delon C, Brown KF, Payne NWS, Kotrotsios Y, Vernon S, Shelton J. Differences in cancer incidence by broad ethnic group in England, 2013-2017. Br J Cancer. 2022;126(12):1765–73. 35233092 10.1038/s41416-022-01718-5 PMC9174248

[24] Brown KF, Rumgay H, Dunlop C, Ryan M, Quartly F, Cox A, et al. The fraction of cancer attributable to modifiable risk factors in England, Wales, Scotland, Northern Ireland, and the United Kingdom in 2015. Br J Cancer. 2018;118(8):1130–41. 29567982 10.1038/s41416-018-0029-6 PMC5931106

[25] Roy S, Vallepu S, Barrios C, Hunter K. Comparison of comorbid conditions between cancer survivors and age-matched patients without cancer. J Clin Med Res. 2018;10(12):911–9. 30425764 10.14740/jocmr3617w PMC6225860

[26] Bluethmann SM, Mariotto AB, Rowland JH. Anticipating the “silver tsunami”: prevalence trajectories and comorbidity burden among older cancer survivors in the United States. Cancer Epidemiol Biomarkers Prev. 2016;25(7):1029–36. 27371756 10.1158/1055-9965.EPI-16-0133 PMC4933329

[27] Eytan DF, Blackford AL, Eisele DW, Fakhry C. Prevalence of comorbidities among older head and neck cancer survivors in the United States. Otolaryngol Head Neck Surg. 2019;160(1):85–92. 30252608 10.1177/0194599818796163

[28] Health survey for England, 2021 part 2. NHS Digital; 2021, 16/05/2023. from https://digital.nhs.uk/data-and-information/publications/statistical/health-survey-for-england/2021-part-2/.

[29] Cancer treatments 2013–2022 NHS Digital: NDRS; 2025 [Available from: https://nhsd-ndrs.shinyapps.io/cancer_treatments/.

[30] Charlson ME, Pompei P, Ales KL, MacKenzie CR. A new method of classifying prognostic comorbidity in longitudinal studies: development and validation. J Chronic Dis. 1987;40(5):373–83. 3558716 10.1016/0021-9681(87)90171-8

[31] UK Data Service. GeoConvert 2024 [Available from: https://geoconvert.ukdataservice.ac.uk/.

[32] The English Index of Multiple Deprivation (IMD) 2015 – guidance. 2015. from https://assets.publishing.service.gov.uk/media/5a7f0e5ded915d74e33f410b/English_Index_of_Multiple_Deprivation_2015_-_Guidance.pdf.

[33] CAS-SOP v4.9.1 Linking treatment tables: chemotherapy, tumour resections, and radiotherapy: NHS Digital; 2024 [Available from: https://digital.nhs.uk/ndrs/data/data-outputs/cancer-data-hub/cancer-treatments.

[34] Schwabish J, Feng A. Considerations for ensuring data aggregation is as inclusive as possible: Urban Institute; 2022 [Available from: https://www.urban.org/urban-wire/considerations-ensuring-data-aggregation-inclusive-possible.

[35] Lewis JH, Kilgore ML, Goldman DP, Trimble EL, Kaplan R, Montello MJ, et al. Participation of patients 65 years of age or older in cancer clinical trials. J Clin Oncol. 2003;21(7):1383–9. 12663731 10.1200/JCO.2003.08.010

[36] Sedrak MS, Freedman RA, Cohen HJ, Muss HB, Jatoi A, Klepin HD, et al. Older adult participation in cancer clinical trials: a systematic review of barriers and interventions. CA Cancer J Clin. 2021;71(1):78–92. 33002206 10.3322/caac.21638 PMC7854940

[37] Miyagi H, Bozorgmehri S, Batra NV, Chatzkel JA, Ramnaraign BH, Hitchcock K, et al. Gender disparities in the clinical trials and real-world utilization of systemic therapy in the management of urothelial carcinoma. JU Open Plus. 2023;1(11):e00059.

[38] Remolina Y, Caballero-Landinez R, Bourlon M. Women with bladder cancer: an underserved population due to a faulty health care system or in a biological disadvantage? Rev Mex Urol. 2022;81:1–11.

[39] Raleigh V, Holmes J. The health of people from ethnic minority groups in England: The Kings Fund; 2021 [Available from: https://www.kingsfund.org.uk/publications/health-people-ethnic-minority-groups-england.

[40] Szczepura A. Access to health care for ethnic minority populations. Postgrad Med J. 2005;81(953):141–7. 15749788 10.1136/pgmj.2004.026237 PMC1743229

[41] Buttery SC, Philip KEJ, Alghamdi SM, Williams PJ, Quint JK, Hopkinson NS. Reporting of data on participant ethnicity and socioeconomic status in high-impact medical journals: a targeted literature review. BMJ Open. 2022;12(8):e064276. 35977760 10.1136/bmjopen-2022-064276 PMC9389090

[42] Drummond R. How ethnicity recording differs across health data sources and the impact on analysis: Office for National Statistics; 2023 [Available from: https://blog.ons.gov.uk/2023/01/16/how-ethnicity-recording-differs-across-health-data-sources-and-the-impact-on-analysis/.

[43] Unger JM, Gralow JR, Albain KS, Ramsey SD, Hershman DL. Patient income level and cancer clinical trial participation: a prospective survey study. JAMA Oncol. 2016;2(1):137–9. 26468994 10.1001/jamaoncol.2015.3924 PMC4824189

[44] Silver D, Blustein J, Weitzman BC. Transportation to clinic: findings from a pilot clinic-based survey of low-income suburbanites. J Immigr Minor Health. 2012;14(2):350–5. 22512007 10.1007/s10903-010-9410-0

[45] Unger JM, Moseley AB, Cheung CK, Osarogiagbon RU, Symington B, Ramsey SD, et al. Persistent disparity: socioeconomic deprivation and cancer outcomes in patients treated in clinical trials. J Clin Oncol. 2021;39(12):1339–48. 33729825 10.1200/JCO.20.02602 PMC8078474

[46] Noor AM, Sarker D, Vizor S, McLennan B, Hunter S, Suder A, et al. Effect of patient socioeconomic status on access to early-phase cancer trials. J Clin Oncol. 2013;31(2):224–30. 23213088 10.1200/JCO.2012.45.0999

[47] Rae S, Shaya S, Taylor E, Hoben J, Oluwashegun D, Lowe H, et al. Social determinants of health inequalities in early phase clinical trials in Northern England. Br J Cancer. 2024;131(4):685–91. 38914804 10.1038/s41416-024-02765-w PMC11333496

[48] Payments and incentives in research: Health Research Authority; 2024 [Available from: https://www.hra.nhs.uk/about-us/committees-and-services/nreap/payments-and-incentives-research/.

[49] Payment guidance for members of the public considering involvement in research: National Institute for Health and Care Research (NIHR); 2024 [Available from: https://www.nihr.ac.uk/payment-guidance-members-public-considering-involvement-research.

[50] Synesi G, Lewis R, Kilburn L, Bliss J, Cheung KC, Gribble H, et al. Reviewing inclusivity of the UK bladder and head and neck oncology trial portfolio through eligibility criteria: a scoping review. Trials. 2026;27(1):19. 41508127 10.1186/s13063-026-09424-w PMC12790708

