# Supplementary material for: Identifying underrepresented groups in oncology clinical trials using routinely collected data in an English academic trial setting
Source: Trials. 2026 May 23;27:504. doi: 10.1186/s13063-026-09812-2 (PMC13377768; doi:10.1186/s13063-026-09812-2)
Supplement: Supplementary file 2 — Supplementary Material 2. [file 13063_2026_9812_MOESM2_ESM.pdf]

## Appendix 1: Characteristics of ICR-CTSU trials included in this study

Table 1: ICR-CTSU bladder cancer trials

| Trial | Participants (n) | Recruitment | Allocated treatment modality | Disease Stage |
|---|---|---|---|---|
| **BC-2001:** Radiotherapy with or without Chemotherapy in Muscle-Invasive Bladder Cancer **(1)** | 444 | 2001-2008 | Chemoradiotherapy | Localised MIBC |
| **BOXIT:** A Randomised phase III placebo-controlled trial evaluating the addition of celecoxib to standard treatment of transitional cell carcinoma of the bladder **(2)** | 462 | 2007-2012 | Resection | Intermediate/high risk of recurrence NMIBC |
| **CALIBER:** Phase II randomised feasibility study of chemoresection and surgical management in low risk, non-muscle-invasive bladder cancer **(3)** | 82 | 2015-2017 | Resection | Recurrent low-risk NMIBC |
| **HYBRID:** A multicentre, randomised, phase II study of hypofractionated bladder radiotherapy with or without image guided adaptive planning **(4)** | 58 | 2014-2015 | Chemoradiotherapy | Localised MIBC |
| **RAIDER:** A randomised phase II trial of adaptive image guided standard or dose escalated tumour boost radiotherapy in the treatment of transitional cell carcinoma of the bladder **(5)** | 291 | 2015-2021 | Chemoradiotherapy | Localised MIBC |
| **SPARE:** Randomised trial of selective bladder preservation against radical excision (cystectomy) in muscle invasive T2/T3 transitional cell carcinoma of the bladder **(6)** | 45 | 2007-2010 | Chemoradiotherapy + resection | MIBC |

Table 1: Characteristics of individual ICR-CTSU bladder cancer trials. n participants = those residing in England and used for analysis only.

Table 2: ICR-CTSU head and neck cancer trials

| Trial | Participants (n) | Recruitment | Allocated treatment modality | Subsite |
|---|---|---|---|---|
| **ART-DECO:** A randomised, multicentre accelerated radiotherapy study of dose escalated intensity-modulated radiotherapy versus standard dose intensity-modulated radiotherapy in patients receiving treatment for locally advanced laryngeal and hypopharyngeal cancers **(7)** | 228 | 2011-2015 | Chemoradiotherapy | Localised laryngeal and hypopharyngeal |
| **COSTAR:** A Multicentre Randomised Study of Cochlear Sparing Intensity Modulated Radiotherapy Versus Conventional Radiotherapy in Patients with Parotid Tumours **(9)** | 97 | 2008-2013 | Radiotherapy | Parotid gland |
| **DARS:** A phase III, randomised, multicentre study of dysphagia optimised intensity modulated radiotherapy (Do-IMRT) versus standard intensity modulated radiotherapy (S-IMRT) in head and neck cancer **(8)** | 87 | 2016-2018 | Chemoradiotherapy | Localised oropharyngeal and hypopharyngeal |
| **INOVATE:** Investigation of novel plasma Human Papilloma Virus DNA assay for treatment response estimation in head and neck cancer **(9)** | 153 | 2020-2022 | Chemoradiotherapy | HPV+ oropharyngeal |
| **PARSPORT:** A multi-centred randomised study of parotid sparing intensity modulated radiotherapy (IMRT) to reduce xerostomia and increase quality of life in head and neck cancer **(10)** | 94 | 2003-2007 | Radiotherapy | Parotid gland |
| **TORPEdO:** A phase III trial of intensity-modulated proton beam therapy versus intensity-modulated radiotherapy for multi-toxicity reduction oropharyngeal cancer **(11)** | 194 | 2020-2023 | Chemoradiotherapy | Localised oropharyngeal |

Table 2: Characteristics of individual ICR-CTSU head and neck cancer trials. n participants = those residing in England and used for analysis only.

Reference list

1. James ND, Hussain SA, Hall E, Jenkins P, Tremlett J, Rawlings C, et al. Radiotherapy with or without chemotherapy in muscle-invasive bladder cancer. N Engl J Med. 2012;366(16):1477-88.
2. Kelly JD, Tan WS, Porta N, Mostafid H, Huddart R, Protheroe A, et al. BOXIT-A Randomised Phase III Placebo-controlled Trial Evaluating the Addition of Celecoxib to Standard Treatment of Transitional Cell Carcinoma of the Bladder (CRUK/07/004). Eur Urol. 2019;75(4):593-601.
3. Mostafid AH, Porta N, Cresswell J, Griffiths TRL, Kelly JD, Penegar SR, et al. CALIBER: a phase II randomized feasibility trial of chemoablation with mitomycin-C vs surgical management in low-risk non-muscle-invasive bladder cancer. BJU Int. 2020;125(6):817-26.
4. Hafeez S, Patel E, Webster A, Warren-Oseni K, Hansen V, McNair H, et al. Protocol for hypofractionated adaptive radiotherapy to the bladder within a multicentre phase II randomised trial: radiotherapy planning and delivery guidance. BMJ Open. 2020;10(5):e037134.
5. Hafeez S, Webster A, Hansen VN, McNair HA, Warren-Oseni K, Patel E, et al. Protocol for tumour-focused dose-escalated adaptive radiotherapy for the radical treatment of bladder cancer in a multicentre phase II randomised controlled trial (RAIDER): radiotherapy planning and delivery guidance. BMJ Open. 2020;10(12):e041005.
6. Huddart R, Birtle A, Lewis R, Bahl A, Falconer A, Maynard L, et al. Results of the SPARE Feasibility Study – Selective Bladder Preservation Against Radical Excision in Muscle Invasive T2/T3 Transitional Cell Carcinoma of the Bladder (CRUK/07/011). International Journal of Radiation Oncology*Biology*Physics. 2012;84(3, Supplement):S119-S20.
7. Nutting CM, Griffin CL, Sanghera P, Foran B, Beasley M, Bernstein D, et al. Dose-escalated intensity-modulated radiotherapy in patients with locally advanced laryngeal and hypopharyngeal cancers: ART DECO, a phase III randomised controlled trial. Eur J Cancer. 2021;153:242-56.
8. Petkar I, Rooney K, Roe JW, Patterson JM, Bernstein D, Tyler JM, et al. DARS: a phase III randomised multicentre study of dysphagia-optimised intensity- modulated radiotherapy (Do-IMRT) versus standard intensity- modulated radiotherapy (S-IMRT) in head and neck cancer. BMC Cancer. 2016;16(1):770.
9. Bhide S, Cheang M. A study to investigate HPV-detect, a new way to measure the response to treatment in patients with Human Papilloma Virus (HPV) positive head and neck cancer: ISRCTN; 2019 [Available from: https://doi.org/10.1186/ISRCTN32335415.
10. Nutting CM, Morden JP, Harrington KJ, Urbano TG, Bhide SA, Clark C, et al. Parotid-sparing intensity modulated versus conventional radiotherapy in head and neck cancer (PARSPORT): a phase 3 multicentre randomised controlled trial. Lancet Oncol. 2011;12(2):127-36.
11. Price J, Hall E, West C, Thomson D. TORPEdO – A Phase III Trial of Intensity-modulated Proton Beam Therapy Versus Intensity-modulated Radiotherapy for Multi-toxicity Reduction in Oropharyngeal Cancer. Clinical Oncology. 2020;32(2):84-8.
