# Supplementary material for: Identifying underrepresented groups in oncology clinical trials using routinely collected data in an English academic trial setting
Source: Trials. 2026 May 23;27:504. doi: 10.1186/s13063-026-09812-2 (PMC13377768; doi:10.1186/s13063-026-09812-2)
Supplement: Supplementary file 3 — Supplementary Material 3. [file 13063_2026_9812_MOESM3_ESM.pdf]

UK phase II and III randomised controlled bladder and head & neck oncology clinical trials: demographic data collection survey

This survey is part of a scoping review conducted by GS for her PhD project. The aim is to ascertain which demographic data were collected from participants of eligible academic trials.

---

1. Which trial is your response regarding? (acronym is sufficient) *

2. Which disease site is your trial in? *

- ○ Bladder cancer
- ○ Head and neck cancer

Were the following demographic factors collected in your trial?

Please note: you can give further details on the next page if you would like

3. Date of birth (age) *

- ◯ Yes
- ◯ No

4. Postcode *

- ◯ Yes
- ◯ No

5. Biological sex (i.e. male or female) *

- ◯ Yes
- ◯ No

6. Gender identity (e.g. man, woman, non-binary) *

- ◯ Yes
- ◯ No

7. Sexual orientation *

- ◯ Yes
- ◯ No

8. Caregiving responsibilities *

- ◯ Yes
- ◯ No

9. Ethnicity *

- ◯ Yes
- ◯ No

10. Religion *

- ◯ Yes
- ◯ No

11. Languages spoken/preferred language *

- ◯ Yes
- ◯ No

12. Employment status (e.g. employed, retired, student) *

- ◯ Yes
- ◯ No

13. Occupation (specific role or generic) *

- ◯ Yes
- ◯ No

14. Income *

- ◯ Yes
- ◯ No

15. Qualifications/education status (e.g. whether they have been to university) *

- ◯ Yes
- ◯ No

16. Smoking status/history *

- ◯ Yes
- ◯ No

17. Alcohol consumption/history *

- ◯ Yes
- ◯ No

18. Co-morbid conditions *

- ◯ Yes
- ◯ No

19. Did you collect any other demographic data? If yes, please specify

